# IMPACT OF ICT AND SOCIAL NETWORKS SITES UTILIZATION ON OLDER PEOPLE’S WELL-BEING AND LONELINESS: A SCOPING REVIEW

**DOI:** 10.1093/geroni/igz038.1949

**Published:** 2019-11-08

**Authors:** Daniele Zaccaria, Georgia Casanova, Antonio Guaita

**Affiliations:** Golgi Cenci Foundation, Abbiategrasso, Italy

## Abstract

In the last decade the debate on ageing issues has been powered by the relationship between older people wellbeing, loneliness, Information Communication Technology (ICT) and Social Networks Sites (SNSs). A scoping review on social experiment studies, analysing the casual effect of technologies use on older adults’ wellbeing and loneliness, has been realized to support the randomized controlled trial included in “Aging in a Networked Society” project. The study aims to review the social experiments on the relationship between technology-use, older people wellbeing and loneliness, to provide a critical analysis of studies, to underline drivers and barriers in existing literature and to provide recommendations for future study and policy. 133 papers have been selected using interdisciplinary search engines (Scopus, Pubmed, Web of sciences, Google Scholar), taking into account contents and methods used. An in-depth examination of 9 experiences of social experiment have been provided, focused on six dimensions: “aims”, “design”, “sampling”, “intervention”, “findings” and “limitations". The literature show the positive effects of ICT, internet and SNSs use on older people wellbeing and quality of life. Our review underlines how the older people shown interest in ICT use to support their social relations, and how it should become a social driver. The low widespread of experimental studies impacts on the literature evidences. The main limits are related to the recruitment and sampling. Social experiment practices, based on controlled randomized trial, should be widespread to better support the evidences in ageing issue.

